# *Vibrio* and Bacterial Communities Across a Pollution Gradient in the Bay of Bengal: Unraveling Their Biogeochemical Drivers

**DOI:** 10.3389/fmicb.2020.00594

**Published:** 2020-04-15

**Authors:** Germán A. Kopprio, Sucharit B. Neogi, Harunur Rashid, Cecilia Alonso, Shinji Yamasaki, Boris P. Koch, Astrid Gärdes, Rubén J. Lara

**Affiliations:** ^1^Department of Chemical Analytics and Biogeochemistry, Leibniz-Institute of Freshwater Ecology and Inland Fisheries, Berlin, Germany; ^2^Tropical Marine Microbiology, Leibniz Centre for Tropical Marine Research, Bremen, Germany; ^3^Marine Biogeochemistry, Instituto Argentino de Oceanografía, Consejo Nacional de Investigaciones Científicas y Técnicas – Universidad Nacional del Sur, Bahía Blanca, Argentina; ^4^Graduate School of Life and Environmental Sciences, Osaka Prefecture University, Izumisano, Japan; ^5^Department of Fisheries Management, Bangladesh Agricultural University, Mymensingh, Bangladesh; ^6^Microbial Ecology of Aquatic Systems, Centro Universitario Región Este, Universidad de la República, Rocha, Uruguay; ^7^Ecological Chemistry, Alfred Wegener Institute, Helmholtz Centre for Polar and Marine Research, Bremerhaven, Germany

**Keywords:** ^15^N depletion, *Arcobacter*, *Cloacibacterium*, organic matter, sewage, isotopes, *Vibrio cholerae*, 16S rRNA diversity

## Abstract

The highly populated coasts of the Bay of Bengal are particularly vulnerable to water-borne diseases, pollution and climatic extremes. The environmental factors behind bacterial community composition and *Vibrio* distribution were investigated in an estuarine system of a cholera-endemic region in the coastline of Bangladesh. Higher temperatures and sewage pollution were important drivers of the abundance of toxigenic *Vibrio cholerae*. A closer relation between non-culturable *Vibrio* and particulate organic matter (POM) was inferred during the post-monsoon. The distribution of operational taxonomic units (OTUs) of *Vibrio* genus was likely driven by salinity and temperature. The resuspension of sediments increased *Vibrio* abundance and organic nutrient concentrations. The δ^13^C dynamic in POM followed an increasing gradient from freshwater to marine stations; nevertheless, it was not a marker of sewage pollution. Bacteroidales and culturable coliforms were reliable indicators of untreated wastewater during pre and post-monsoon seasons. The presumptive incorporation of depleted-ammonium derived from ammonification processes under the hypoxic conditions, by some microorganisms such as *Cloacibacterium* and particularly by *Arcobacter* nearby the sewage discharge, contributed to the drastic ^15^N depletion in the POM. The likely capacity of extracellular polymeric substances production of these taxa may facilitate the colonization of POM from anthropogenic origin and may signify important properties for wastewater bioremediation. Genera of potential pathogens other than *Vibrio* associated with sewage pollution were *Acinetobacter*, *Aeromonas*, *Arcobacter*, and *Bergeyella*. The changing environmental conditions of the estuary favored the abundance of early colonizers and the island biogeography theory explained the distribution of some bacterial groups. This multidisciplinary study evidenced clearly the eutrophic conditions of the Karnaphuli estuary and assessed comprehensively its current bacterial baseline and potential risks. The prevailing conditions together with human overpopulation and frequent natural disasters, transform the region in one of the most vulnerable to climate change. Adaptive management strategies are urgently needed to enhance ecosystem health.

## Introduction

The genus *Vibrio* comprises a diverse group of more than 140 species, within which *V. cholerae*, *V. parahaemolyticus*, *V. vulnificus*, *V. fluvialis*, and *V. mimicus* are etiological agents of human and animal disease causing gastroenteritis, sepsis and necrosis. Toxigenic and non-toxigenic populations of *Vibrio* species coexist generally in the aquatic environment. *Vibrio cholerae* is a natural inhabitant of estuaries and some strains are responsible of cholera disease. Cholera gravis cases are characterized by profuse watery diarrhea which leads to a life-threatening dehydration (Sack et al., 2004). Recent outbreaks in developing countries, particularly after natural disasters, classified cholera as a re-emerging disease (Morens et al., 2004; Vouga and Greub, 2016). Notably, the low-lying coastal areas of the Bay of Bengal are not only claimed as an endemic region for the pandemic serogroups of *V. cholerae* but also as the pivotal base of the global spread of cholera (Mutreja et al., 2011).

Cholera toxin and toxin co-regulated pilus are the main virulence factors of the pandemic serogroups of *V. cholerae*: O1 and O139. In endemic areas, the incidence of cholera in human populations is coupled with the distribution of *V. cholerae* in aquatic ecosystems. Temperature, salinity and plankton abundance are important factors explaining the distribution of *V. cholerae* (Huq et al., 2005; Constantin de Magny et al., 2008). Under adverse environmental conditions, *V. cholerae* is usually detected in a non-culturable state of reduced metabolism but with the potential to grow exponentially under favorable settings. In the last years, organic matter and suspended sediments were identified as key drivers for the distribution of *Vibrio* species (Lara et al., 2009; Neogi et al., 2018). Since only a few studies offer scattered information about these drivers, more investigations are needed for an integral assessment of the biogeochemical factors behind *Vibrio* ecology.

*Vibrio* species tend to form biofilms in seston and present a wide variety of enzymes to degrade and assimilate organic matter playing unique roles in biogeochemical cycles (Lara et al., 2011; Neogi et al., 2018; Liang et al., 2019). Moreover, *Vibrio* populations interact with other microorganisms during the process of nutrient remineralization, which is performed by a variety of microbial taxa with different metabolic and physiological capabilities. The precise way in which organic and inorganic nutrients influence dynamics of aquatic bacterial populations remains largely unresolved (Bunse and Pinhassi, 2017). Biological interactions are emerging as a key factor driving bacterial communities and many virulence features of *V. cholerae* evolved not only in response to environmental factors but also to biotic pressures (Schwartz et al., 2019). Studies combining the ecology of *V. cholerae* and other *Vibrio* populations in relation with bacterial community composition are urgently needed in cholera endemic areas.

The coastline of the Bengal delta is largely influenced by monsoon rainfalls, recurrent climatic disasters and pollutants. Among the diverse groups of bacteria existing in coastal waters, some can respond quickly to changing environmental conditions and are reliable indicators of hydrological changes and water pollution (Cao et al., 2017; Kegler et al., 2017). Sewage impacts on human and ecosystem health by eutrophication, spread of water-borne diseases, and as a source of heavy metals and persistent organic pollutants (reviewed by Schwarzenbach et al., 2010). Stable isotopes of nitrogen and carbon are other indicators of pollution and the origin of the organic matter. An elevated signature of δ^15^N in the suspended particulate matter is generally a marker of anthropogenic impacts; however, recent studies evidenced the marked depletion of ^15^N in heavily polluted systems (Ke et al., 2017; Kopprio et al., 2018; Hong et al., 2019). The combination of bacterial and isotopic indicators offers a tool of higher resolution in ecological studies of coastal systems.

Little work has been performed to understand the effect of the composition and distribution of organic matter influencing the population dynamics of *Vibrio* species along with the changes in coexisting estuarine bacterial communities in cholera endemic regions. Therefore, this study systematically explored a polluted estuary in Bangladesh with the following aims: (1) to elucidate the role of water quality and nutrient biogeochemistry on the dynamic of *Vibrio* species and culturable bacteria; (2) to study the seasonal distribution of bacterial communities across the estuarine and pollution gradient as well as their influence on the isotopic signature of the particulate organic matter; and (3) to identify potential pathogens and key bacteria for biogeochemical processes and bioremediation. We hypothesized a strong influence of sewage and sediment resuspension in the distribution of toxigenic *V. cholerae*, *Vibrio* species and genera of potential pathogens. Other global hypothesis was that the specific effects of sewage input and sediment resuspension on aquatic bacterial community and organic matter composition are clearly discernible. The integrated assessment of hydrological changes and pollution on the dynamic of biogeochemical and bacterial markers influencing ecosystem health enhances the multidisciplinary scope of this study.

## Materials and Methods

### Study Site

**FIGURE 1:**
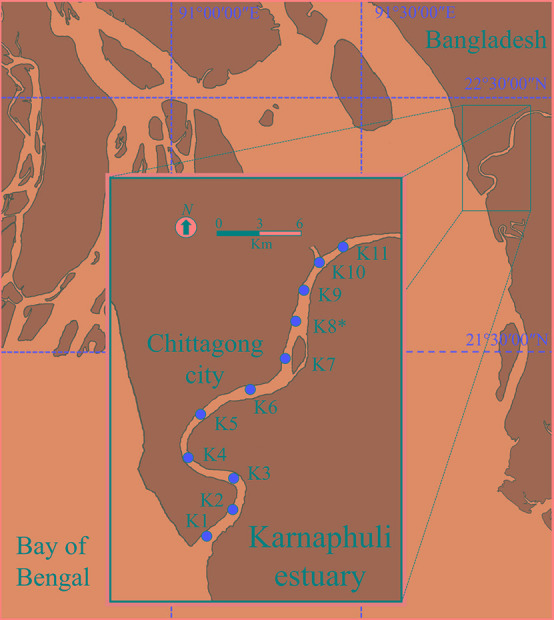
Location of the Karnaphuli estuary and sampling stations in the Bay of Bengal. (*) strongly influenced by sewage pollution.

The Karnaphuli estuary is characterized by semidiurnal tides and flows across the southern banks of the Chittagong port city into the east coastline of the Bay of Bengal (Figure 1). In the last decades, Chittagong metropolitan area has experienced a considerable population growth and nowadays comprises of more than 4 million inhabitants. As it occurs in most of the coastal cities of Bangladesh, Chittagong has insufficient water treatment facilities and the effluents from diverse sources are discharged practically raw in the estuary endangering ecosystem and public health (Wang et al., 2016). The lack of safe drinking water and sanitation, and the prevalence of water-borne diseases are key challenges for Chittagong’s authorities and stakeholders (Lara et al., 2009). In the last decades the region experienced strong cyclones, which killed thousands of people and produced severe floods.

### Sampling Strategy

The Karnaphuli estuary was sampled at 11 stations (Figure 1) during pre and post-monsoon seasons (May 15^th^ and December 8^th^ of 2016, respectively). The pre-monsoon campaign was carried out 3 days before the cyclone Roanu, which forced half a million people to leave their homes and caused some fatalities. Water and Suspended Particulate Matter (SPM) were sampled with a motor boat across a ∼20 Km transect, starting at station 1 (K1) with marine water and finishing at K11 with freshwater. The station with a strong influence of the sewage discharge of Chittagong city corresponded to K8. To separate larger aggregates and organisms from nanoplankton, water was fractionated with a net (20 μm pore size) at K1, K3, K5, K7, and K11. To simulate the likely effect of water runoff, coastal sediments were resuspended in a known volume of estuarine water (∼2 g L^–1^) at K3, K5, K8 and K11.

### *In situ* Measurements and Preparations

Temperature, salinity, conductivity, pH and dissolved oxygen were measured *in situ* with a multiparametric meter WTW 3430 (Xylem Analytics). Water and SPM were collected at 30 cm below the surface and stored in clean and sterile 2 L plastic bottles. The nanoplankton fraction (SPM < 20 μm) and SPM enriched with coastal sediments (SPM-Sed) were sampled at the same depth and stored in bottles with the mentioned characteristics. Water for dissolved nutrients was filtered with a Syringe filter Minisart PES High Flow (Sartorius) and stored in chemically clean HDPE 50 mL bottles. For Catalyzed Reporter Deposition – Fluorescence *In Situ* Hybridization (CARD-FISH) studies, water samples were fixed with buffered particle-free paraformaldehyde (1% final concentration) and kept in PE 50 mL bottles. All samples were transported in insulated plastic boxes and processed within 6 h.

### Biogeochemical Analyses

Bottles for dissolved nutrients were frozen at −20°C until further analyses. All samples were filtered through precombusted GF/F glass-fiber filters (Whatman). Filters for chlorophyll determinations were preserved frozen at −20°C and those for particulate organic matter (POM) and stable isotope measurements were dried overnight at 50°C and stored in a vacuum desiccator. Dissolved inorganic nutrients were determined according to standard methods with an auto-analyzer (Evolution III, Alliance Instrument). Dissolved organic carbon (DOC) was measured by high temperature catalytic oxidation with a Shimadzu TOC-VCPN analyzer and total dissolved nitrogen (TDN) was measured simultaneously by chemiluminescence detection with a Shimadzu TNM-1. Dissolved organic nitrogen (DON) was calculated by difference as DON = TDN – (ammonium + nitrate + nitrite). Pigments were extracted in ethanol ≥99.5% (Roth) and an overnight incubation at 4°C in the darkness. Chlorophyll *a* content was estimated photometrically after Marker et al. (1980).

All dried filters were acidified (1N HCl) to remove inorganic carbon and placed in tin or silver capsules for particulate organic nitrogen and carbon (PON and POC) determination, respectively. The capsules were oxidized at 1000°C under pure oxygen in an elemental analyzer (EURO EA, HEKAtech). Carbon and nitrogen stable isotopes (^13^C and ^15^N) were measured with a mass spectrometer (Thermo Finnigan Delta Plus, Thermo Fisher Scientific) coupled with an elemental analyzer (Flash EA 1112, Thermo Fisher Scientific). Acetanilide (HEKAtech) and peptone were used as internal standards. Stable isotopes were reported as delta (δ) values in parts per thousand (denoted as ‰), carbon relative to Pee Dee Belemnite and nitrogen relative to nitrogen in air. The isotope ratios were measured in accordance with the following reference standards: IAEA-N-1, IAEA-N-2, NBS 22, and USGS-24.

### Culturable Bacteria

Water was directly spread or placed after filtration through sterile mixed cellulose membranes of 0.45 μm pore size (ME 25, Whatman) on Thiosulfate Citrate Bile salts Sucrose (TCBS, Roth) agar plates. Colony Forming Units (CFUs) of presumptive *Vibrio* were counted after overnight incubation at 37°C. Water (25 mL) was enriched in Alkaline Peptone Water (APW, Roth) and incubated overnight at the same temperature. To estimate the colonies of *V. cholerae* growing in TCBS, 30 presumptive colonies were isolated and enriched in APW. Water was also spread in duplicate directly or after serial dilutions in 1× phosphate buffered saline (PBS) on Mac Conkey agar (Roth) for the determination of coliforms and on Plate Counting Agar (PCA, Roth) for terrestrial heterotrophic bacteria. CFUs were counted after overnight incubation at 37°C and 30°C, respectively.

## Card-Fish

Samples for CARD-FISH were kept overnight at 4°C and known volumes (10–50 mL) were filtered through GTTP Isopore membranes (Merck Millipore) of 0.2 μm pore size, and stored in sterile Petri dishes at −20°C. The protocol for CARD-FISH was performed basically after Pernthaler et al. (2002). Briefly, cells were permeabilized with lysozyme (Sigma Aldrich) solution (10 mg mL^–1^) and endogenous peroxidases were inactivated with HCl (0.01 N). The Horseradish Peroxidase (HRP) – labeled probes (Biomers.net) used for hybridization were: Vchomim1276 for *Vibrio cholerae* and *V. mimicus* and *Vibrio*-GV for *V. parahaemolyticus*, *V. vulnificus* and several other *Vibrio* species excluding *V. cholerae* (Table 1). The HRP-probes (50 ng μL^–1^) were mixed in a relation 1:100 with the hybridization buffer containing 50% formamide for Vchomim1276 and 30% for *Vibrio*-GV. After an incubation of 2 h at 35°C, samples were rinsed in the corresponding washing buffer and subsequently in 1× PBS. The CARD step was performed in the darkness for 15 min at 37°C, with a tyramide-containing amplification buffer (1 μg μL^–1^ tyramide conjugated with Alexa 488, Thermo Fisher Scientific). Filters were counterstained with DAPI (1 μg mL^–1^) and cells were counted across 50 random grids (120 × 120 μm). The abundance (cells mL^–1^) was calculated considering the total surface and volume filtered.

**TABLE 1:** Primers and probes used for catalysed reporter deposition – fluorescence *in situ* hybridization (CARD-FISH), quantitative polymerase chain reaction (qPCR) and 16S rRNA studies.

**Gene or target**	**Primer or probe**	**Sequence (5′ – 3′)**	**Source**
**For CARD-FISH**			
*Vibrio cholerae* – *V. mimicus*	Vchomim1276	HRP-ACT TTG TGA GAT TCG CTC CAC CTC G	Grim et al., 2009
*Vibrio* species	*Vibrio*-GV	HRP-AGG CCA CAA CCT CCA AGT AG	Giuliano et al., 1999
**For qPCR**			
*ompW* – *V. cholerae*	*ompW*-F1	AAG CTC CGC TCC TGT ATT TGC	modified from
	*ompW*-F2	ACT AGC CGC TCC TGT ATT TGC	Bliem et al., 2015
	*ompW*-R	GCT ATT AAC TGC CAA CTC ACT TTG AG	
	*ompW*-FAM	FAM-CAC CAA GAA GGT GAC TTT-BMN-Q535	
*ctxA – V. cholerae*	*ctxA*-F	GCA TAG AGC TTG GAG GGA AGA G	modified from
	*ctxA*-R	CAT CGA TGA TCT TGG AGC ATT C	Bliem et al., 2015
	*ctxA*-HEX	HEX-CAT CAT GCA CCG CCG-BMN-Q535	
Internal control (IC)	IC-F	GAC CAC TAC CAG CAG AAC AC	this study
	IC-F-HEX	HEX-GAC CAC TAC CAG CAG AAC AC-BMN-Q535	
	IC-F-FAM	FAM-GAC CAC TAC CAG CAG AAC AC-BMN-Q535	
	IC-R	GAA CTC CAG CAG GAC CAT G	
**For 16S rRNA**			
V3-V4 regions	341F	CCT ACG GGN GGC WGC AG	Klindworth et al., 2012
	785R	GAC TAC HVG GGT ATC TAA KCC	

*HRP, horseradish peroxidase; *ompW*, outer membrane protein W; *ctxA*, cholerae toxin A; FAM, 6-carboxyfluorescein; BMN-Q535, biomers.net quencher; HEX, 6-hexachloro-fluorescein phosphoramidite.*

### DNA Extraction

Water (200–100 mL) was filtered through the mentioned GTTP membranes (Merck Millipore), stored at −20°C and the DNA was extracted after Boström et al. (2004). Briefly, cells were lysed firstly with lysozyme (1 mg mL^–1^) and then treated with SDS (1%) and proteinase K (Thermo Fisher Scientific, 100 μg mL^–1^). DNA was recovered after incubation with 0.6 volumes of isopropanol (ROTIPURAN, Roth) and two centrifugation steps for 20 min at 20,000 *g* and 4°C. In case of inhibition, the extracts were diluted or cleaned with a genomic DNA clean-up kit (NucleoSpin, Macherey-Nagel). For culturable bacteria, DNA was extracted using a simple boiling method. DNA concentrations were measured in a NanoQuant plate with an infinite M200 PRO Multimode reader (TECAN).

### Quantitative Polymerase Chain Reaction (qPCR)

The detection of *V. cholerae* was based on the qPCR protocol of Bliem et al. (2015). The protocol was modified in two duplex strategies in our Bio-Rad CFX Connect real time system: (1) the *ompW* probe (Table 1) was labeled with 6-carboxyfluorescein (FAM, Biomers.net) and the internal control (IC) with 6-hexachloro-fluorescein phosphoramidite (HEX, Biomers.net); and (2) *ctxA* was labeled with HEX and IC with FAM. For *ompW* detection, all qPCR were performed in a final reaction volume of 20 μL containing 1× SensiFAST Probe Lo-ROX master mix (Bioline), *ompW*-F1 (150 nM), *ompW*-F2 (50 nM), *ompW*-R (200 nM), *ompW*-FAM (200 nM), IC-F (100 nM), IC-R (100 nM), IC-HEX (25 nM), and sample and IC templates. For *ctxA* quantification, the primers were: *ctxA*-F (200 nM), *ctxA*-R (200 nM), *ctxA*-HEX (200 nM), IC-F (100 nM), IC-R (100 nM), IC-FAM (50 nM). The qPCR conditions were 1 min of denaturation at 95°C followed by 45 cycles at 95°C for 10 s and at 60°C for 30 s. Each run contained a dilution series of DNA from *V. cholerae* O1 N 16961 and negative controls. The qPCR reactions were managed and analyzed using the software Bio-Rad CFX Manager 3.0. The genomic units (GU) of *V. cholerae* were calculated based on the genome length and the molar mass of *V. cholerae* O1 N 16961, DNA concentrations and total filtered volume.

### 16S rRNA Diversity

The V3-V4 hypervariable region of the 16S rRNA gene was amplified by the primers Bact-341F and Bact-785R (Table 1) and sequenced by the company LGC Genomics on an Illumina MiSeq platform. After primer removal and demultiplexing, sequences were trimmed using trimmomatic v0.36 (Bolger et al., 2014) and merged with PEAR v0.9.8 (Zhang et al., 2014). Quality trimmed sequences were 54,917 ± 25,626 (mean ± SD) per sample. Clustering of Operational Taxonomic Units (OTUs) was performed with Minimum Entropy Decomposition MED v2.1 (Eren et al., 2015) and the OTU number per sample was 20,676 ± 12,550. For taxonomic classification, OTU representatives were submitted to SilvaNGS (v132^1^) using a threshold of sequence similarity of one for clustering (Quast et al., 2013). Singletons, doubletons, archaea, chloroplasts, mitochondria were removed from the analysis. Demultiplexed and primer-clipped sequences were deposited at the European Nucleotide Archive using the data brokerage service of the German Federation for Biological Data (Diepenbroek et al., 2014) with the accession number PRJEB35775.

### Statistical Analysis

Removal of OTUs with poor alignment quality, pooling of taxa and calculations of abundance and diversity were performed in R v3.5.1 (R Core Team, 2018) and additional packages. Statistics and graphics were carried out with R, XLSTAT (Addinsoft, 2018), PRIMER v6 and Xact 7.21d. Permutational multivariate analysis of variance (PERMANOVA) was used to evaluate differences between seasons and fractions in culturable bacteria and *Vibrio* counts and in main orders, genera and OTUs. In case of significant differences among fractions, pair-wise tests were performed and the variables were compared with Kruskal-Wallis one-way analysis of variance (ANOVA) and Dunn pair-wise comparisons. Two Canonical Correspondence Analyses (CCA 1 and 2) were carried out using the environmental parameters as explanatory variables of the distribution of: CCA 1) Culturable bacteria and *Vibrio* abundance (counts or genomic units L^–1^); and CCA 2) OTU numbers for the main bacterial orders (total n at each order). For the CCAs, the environmental and the biological variables were logarithmical [log (x + 1)] and square-root transformed, respectively. Linearity was checked with the Monte Carlo test after 1000 permutations. Relationships between and within biological and environmental variables were also studied using Pearson correlations (r) based on the log-transformed data. The 70 most abundant OTUs of those genera with a sequence relative abundance higher than 1% at any of the K8 samples were displayed in a heat map, K3 and K11 were included as “control” sites.

## Results

### Distribution and Seasonality of *Vibrio* Across the Estuarine Continuum

The *ompW* gene for *V. cholerae* was positive in all water samples directly enriched with APW and in the 83% of the non-cultured samples. The *ctxA* gene followed the opposite trend, with 25% of the positive cases in the culturable fraction and 38% in the non-enriched cases. The samples derived from the presumptive colonies in TCBS were positive for *ompW* in the 23% of the cases but negative for *ctxA*. The CFUs in TCBS or culturable *Vibrio* counts (CVCs) were significantly positively correlated with *Vibrio*-GV counts (*r* = 0.70, *p* < 0.001) and *Vibrio* OTUs (*r* = 0.55, *p* < 0.001). A total of 74 different OTUs for *Vibrio* were detected.

**FIGURE 2:**
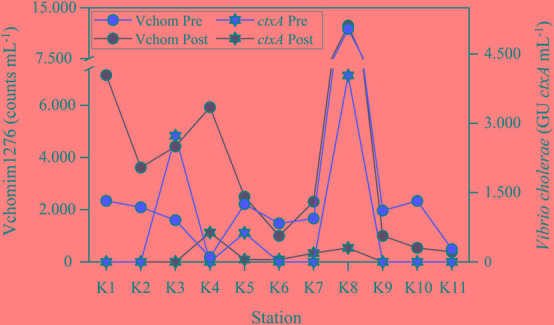
Seasonal abundances of *Vibrio cholerae* and *V. mimicus* based on CARD-FISH counts with the probe Vchomim1276 and genomic units (GU) of cholera toxin gene A (*ctxA*) across the estuarine gradient. Station K8 was strongly influenced by sewage pollution.

The highest number of *ctxA* GU in the SPM was found at the station receiving the sewage discharge (K8) during the warmer season (Figure 2). Other peaks of *ctxA* GU were associated to downstream stations of intermediate salinities. Ammonium, closely related to coliforms, and POC were positively correlated with *ctxA* GU (Table 2). Vchomim1276 counts in SPM presented maximum values at K8 during both seasons (Figure 2) and generally higher values during the post-monsoon. POC showed significant correlations with Vchomim1276 counts and *ompW* GU (Table 2). Salinity as an indicator of the estuarine gradient was positively correlated principally with *Vibrio* OTUs, CVCs and *Vibrio*-GV. Water temperature as an indicator of seasonality was significantly positively correlated with heterotrophic culturable bacteria and CVCs.

**TABLE 2:** Main correlations (Pearson coefficient = r) of salinity, temperature, ammonium and particulate organic carbon (POC) with biological and other environmental variables in the suspended particulate matter (*n* = 22).

	***Vibrio* and culturable bacteria**			**Main orders**			**Environmental variables**		
	**Variables**	***r***	***p***	**Variables**	***r***	***p***	**Variables**	***r***	***p***
Salinity	*Vibrio*-GV	0.79	<0.001	Oceanospirillales	0.94	<0.001	Chlorophyll	–0.82	<0.001
	*Vibrio* OTUs	0.50	0.017	Rhodobacterales	0.91	<0.001	Silicate	–0.77	<0.001
	CVC	0.47	0.028	Chthoniobacterales	–0.90	<0.001	δ^13^C	0.77	<0.001
				Cellvibrionales	0.83	<0.001	Nitrate	0.60	0.004
				Pirellulales	–0.78	<0.001	Ammonium	0.55	0.008
				Vibrionales	0.74	<0.001	pH	0.50	0.019
				Microtrichales	–0.70	<0.001	δ^15^N	0.45	0.039
Temperature	Heterotrophic	0.78	<0.001	n.s.			δ^15^N	0.43	0.048
	CVC	0.58	0.005						
Ammonium	Coliforms	0.80	<0.001	Bacteroidales	0.90	<0.001	Silicate	0.80	<0.001
	*ctxA* GU	0.53	0.010	SAR 11	–0.68	<0.001	Phosphate	0.79	<0.001
				Pseudomonadales	0.64	0.002	DO	–0.78	<0.001
				Campylobacterales	0.60	0.003	pH	–0.77	<0.001
				Oceanospirillales	–0.56	0.007	δ^15^N	–0.73	<0.001
				Vibrionales	–0.51	0.015	PON	0.63	0.002
				Flavobacterales	0.49	0.022	Salinity	–0.55	0.008
POC	Vchomim1276	0.63	0.002	SAR 11	–0.59	0.004	PON	0.97	<0.001
	*ctxA* GU	0.50	0.018	Bacteroidales	0.46	0.032	DOC	0.74	<0.001
	*ompW* GU	0.49	0.019				DO	–0.63	0.002
	Coliforms	0.43	0.044				δ^15^N	–0.63	0.002
							Ammonium	0.53	0.011
							DON	0.51	0.016
							Phosphate	0.49	0.020

*POC, particulate organic carbon; DO, dissolved oxygen; PON, particulate organic nitrogen; DOC, dissolved organic carbon; *Vibrio*-GV, other *Vibrio* species identified with a CARD-FISH probe; OTUs, operational taxonomic units; CVC, culturable *Vibrio* counts; *ctxA* GU, cholera toxin A genomic units; Vchomim1276, *Vibrio cholerae* and *V. mimicus* identified with a CARD-FISH probe*; ompW* GU, outer membrane protein W gene for *V. cholerae* genomic units; n.s., not significant.*

PERMANOVA revealed significant differences between seasons (pseudo-F = 7.28, *p* < 0.001) and fractions (pseudo-F = 3.52, *p* = 0.013). Fractions refer to SPM, SPM < 20 μm and SPM-Sed. No interaction was detected between seasons and fractions (pseudo-F = 0.95, *p* = 0.954). According to pair-wise tests and considering the differences between fractions, SPM-Sed differed significantly from SPM (*t* = 2.20, *p* = 0.013) and SPM < 20 μm (*t* = 2.41, *p* = 0.006). No significant differences were found between SPM and SPM < 20 μm (*t* = 0.91, *p* = 0.451). SPM-Sed (Table 3) shows significantly higher values of Vchomim1276 and *Vibrio*-GV counts, *ompW* GU, DON, POC and PON. No significance differences in any parameter were found between SPM and SPM < 20 μm.

**TABLE 3:** Distribution of *Vibrio* counts, nutrients and stable isotopes (mean value ± standard deviation) in the fractions: suspended particulate matter (SPM), nanoplankton (SPM < 20 μm), and SPM enriched with sediments (SPM-Sed).

**Variables**	**SPM (*n* = 22)**	**SPM < 20 μ m (*n* = 10)**	**SPM-Sed (*n* = 8)**	**K**	**p**
*Vibrio counts*					
CVC (CFU × 10^3^ mL^–1^)	1.3 ± 2.9	1.7 ± 3.4	5.9 ± 11.5	1.7	0.426
Vchomim1276 (cell × 10^3^ mL^–1^)	**3.2 ± 3.4^a^**	**1.6 ± 1.0^a^**	**8.4 ± 5.5^b^**	**11.0**	**0.004**
*Vibrio*-GV (cell × 10^3^ mL^–1^)	**3.3 ± 2.8^a^**	**3.3 ± 3.8^a^**	**16.3 ± 12.0^b^**	**15.0**	**<0.001**
*ompW* (GU × 10^3^ mL^–1^)	**1.7 ± 2.3^a^**	**1.0 ± 0.9^a^**	**26.1 ± 51.5^b^**	**7.0**	**0.029**
*ctxA* (GU × 10^2^ mL^–1^)	3.9 ± 10.0	0.2 ± 0.6	8.4 ± 14.4	4.9	0.087
*Vibrio* (OTUs x 10^2^)	6.0 ± 18.5	3.1 ± 7.6	2.7 ± 7.3	1.1	0.565
*Inorganic nutrients*					
Phosphate (μM)	4.5 ± 7.6	2.3 ± 1.5	4.2 ± 6.0	0.6	0.731
Ammonium (μM)	22.4 ± 40.9	11.4 ± 19.2	41.7 ± 64.6	2.0	0.362
Nitrite (μM)	2.7 ± 2.1	2.3 ± 2.3	2.4 ± 2.1	0.5	0.798
Nitrate (μM)	19.9 ± 10.7	20.6 ± 11.9	16.7 ± 11.8	0.5	0.785
Silicate (μM)	140 ± 63	117 ± 53	178 ± 118	1.4	0.505
*Organic nutrients*					
DOC (μM)	225 ± 100	216 ± 82	366 ± 179	5.3	0.071
DON (μM)	**49.9 ± 172^ab^**	**8.6 ± 4.1^a^**	**124 ± 278^b^**	**7.4**	**0.025**
POC (μM)	**240 ± 188^a^**	**249 ± 165^a^**	**1662 ± 1194^b^**	**17.4**	**<0.001**
PON (μM)	**39.2 ± 34.9^a^**	**42.6 ± 36.3^a^**	**220 ± 155^b^**	**16.5**	**<0.001**
C/N particulates	6.3 ± 1.1	6.1 ± 0.7	7.3 ± 1.5	4.0	0.136
*Stable isotopes*					
δ^15^N (‰)	6.0 ± 2.8	6.0 ± 1.9	5.4 ± 2.8	0.4	0.825
δ^13^C (‰)	−24.6 ± 2.2	−24.4 ± 2.2	−24.8 ± 0.9	2.5	0.288

*Significant differences according to Kruskal-Wallis one-way analysis of variance are printed in bold, superscript letters indicate differences following Dunn pair-wise comparisons. CVC, culturable *Vibrio* counts; CFU, colony forming unit; Vchomim1276, *Vibrio cholerae* and *V. mimicus* identified with a CARD-FISH probe; *Vibrio*-GV, other *Vibrio* species identified with a CARD-FISH probe; *ompW*, outer membrane protein W gene for *V. cholerae*; GU, genomic units by qPCR; *ctxA*, cholera toxin A; OTUs, operational taxonomic units; DOC, dissolved organic carbon; DON, dissolved organic nitrogen; POC, particulate organic carbon; PON, particulate organic nitrogen.*

**FIGURE 3:**
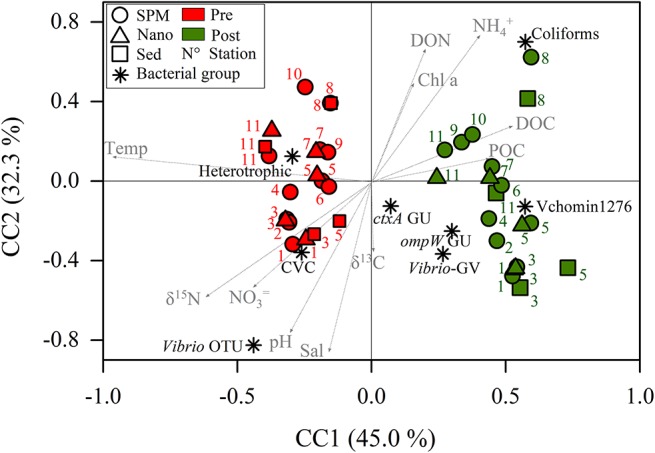
Canonical correspondence analysis (CCA) using environmental parameters as explanatory variables of culturable bacteria and *Vibrio* counts. SPM, suspended particulate matter; Nano, SPM after 20 μm net filtration; Sed, SPM enriched with sediments; Pre, Pre-monsoon; Post, Post-monsoon; Temp, temperature; Sal, salinity; DON, dissolved organic nitrogen; DOC, dissolved organic carbon; Chl *a*, chlorophyll *a*; POC, particulate organic carbon; CVC, culturable *Vibrio* counts; OTU, operational taxonomic units; GU, genomic units by qPCR; *ctxA*, cholera toxin A; *ompW*, outer membrane protein W gene for *Vibrio cholerae*; Vchomim1276, CARD-FISH counts for *V. cholerae* and *V. mimicus*; *Vibrio*-GV; CARD-FISH counts for other *Vibrio* species.

The CCA (Figure 3) ordinated samples and culturable bacteria and *Vibrio* based on the environmental parameters and suggested also seasonal differences. The constrained inertia was 71.6% of the total inertia and the relation among the variables were considered linear (pseudo-F = 1.61, *p* < 0.001). All pre-monsoon samples were located at the negative side of the CC1 (which explains the 45% of the variation) together with culturable *Vibrio*, heterotrophic bacteria and *Vibrio* OTUs. These variables were linked mainly to temperature, δ^15^N and nitrate. At the positive side of CC1 were ordinated all post-monsoon samples together with coliforms and *Vibrio* counts (qPCR and CARD-FISH). Organic matter, ammonium and chlorophyll were also located at the positive side of CC1. At the positive extreme of CC2 (which explains the 32% of the variation) were ordinated the samples of K8 with higher ammonium and DON values and some freshwater samples with elevated chlorophyll values. The samples more influenced by the sea were at the positive extreme of CC2.

### Bacterial Communities: Estuarine Gradient and Pollution Impact

The orders Rhodobacterales and Oceanospirillales presented higher relative sequence abundances at the marine stations (Supplementary Figure S1). The main genera in the Rhodobacterales were *Marivivens* (63% of the total OTUs for the order Rhodobacterales) and *Roseobacter* clade CHAB-I-5 lineage (11%), while in the Oceanospirillales were *Bermanella* (22%), *Oleiphilus* (17%), *Halomonas* (9%), and *Marinomonas* (7%). The relative sequence abundances of Vibrionales (96% *Vibrio*) were also higher at the marine stations and particularly during the Pre-monsoon season (up to 20%, Supplementary Figure S1). Salinity as an indicator of the estuarine gradient was positively correlated with Oceanospirillales, Rhodobacterales, Cellvibrionales and Vibrionales, while negatively mainly with Chthoniobacterales, Pirellulales and Microtrichales (Table 2). The Microtrichales (96% CL500-29 marine group) and Synechococcales (100% *Cyanobium* PCC-6307) were the dominant orders at the freshwater stations.

Other abundant orders at intermediate stations (Supplementary Figure S1) were the Betaproteobacteriales (45% Burkholderiaceae unclassified and 5% *Dechloromonas*) and Micrococcales (70% *Candidatus Aquiluna* and 5% *Candidatus Planktoluna*). The Bacillales (85% *Bacillus*) dominated the samples enriched with sediments at some stations during the post-monsoon. Ammonium was significantly correlated with Bacteroidales, Pseudomonadales, Campylobacterales and Flavobacteriales (Table 2). Moreover, POC was positively correlated with Bacteroidales and coliforms. The principal genera in the order Bacteroidales were *Prevotella* 9 (50%) and *Macellibacteroides* (16%), in Pseudomonadales were *Acinetobacter* (76%) and *Pseudomonas* (23%), in Campylobacterales were *Arcobacter* (93%) and *Sulfurospirillum* (6%), and in Flavobacteriales were *Cloacibacterium* (35%) and *Flavobacterium* (28%).

**FIGURE 4:**
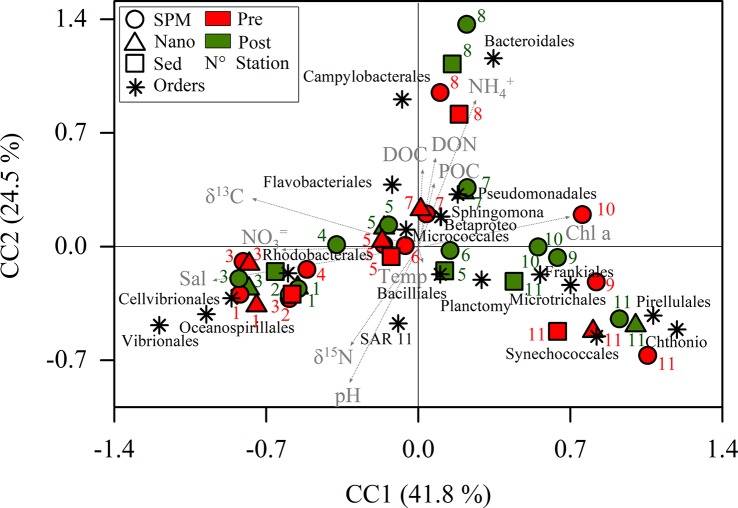
Canonical correspondence analysis (CCA) using environmental parameters as explanatory variables of the main orders. SPM, suspended particulate matter; Nano, SPM after 20 μm net filtration; Sed, SPM enriched with sediments; Pre, Pre-monsoon; Post, Post-monsoon; Temp, temperature; Sal, salinity; DON, dissolved organic nitrogen; DOC, dissolved organic carbon; Chl *a*: chlorophyll *a*; POC, particulate organic carbon; Betaproteo, Betaproteobacteriales; Sphingomonas, Sphingomonadales; Planctomy, Planctomycetales; Chthonio, Chthoniobacterales.

PERMANOVA indicated no significant differences between seasons (pseudo-F = 1.16, *p* = 0.289) and fractions (pseudo-F = 0.994, *p* = 0.407) at order level. At genera level, PERMANOVA did not evidence significant differences between seasons (pseudo-F = 1.95, *p* = 0.103) and fractions (pseudo-F = 0.84, *p* = 0.530). Following the same trend, no significant differences were found between seasons (pseudo-F = 2.1, *p* = 0.054) and fractions (pseudo-F = 0.68, *p* = 0.800) at OTU level. The non-seasonality in the distribution of the main orders was also indicated by the CCA ordination (Figure 4). The constrained inertia was the 72.8% of the total inertia and the relations between the data sets were considered linear (pseudo-F = 4.39, *p* < 0.001). The CC1 (which explained the 41.8% of the variation) ordinated at its negative side Vibrionales, Oceanospirillales, Cellvibrionales and Rhodobacterales. These orders were grouped principally together with salinity, δ^13^C and nitrate. The marine stations (K1, K2 and K3) were located at the negative extreme of CC1. At the positive side were ordinated most of the freshwater stations together with the Chthoniobacterales, Pirellulales, Synechococcales, Microtrichales and Frankiales. These variables were principally associated with chlorophyll *a*. The positive side of CC2 grouped the samples from K8 and K7, principally together with Bacteroidales, Campylobacterales, Flavobacteriales, ammonium and organic matter.

**FIGURE 5:**
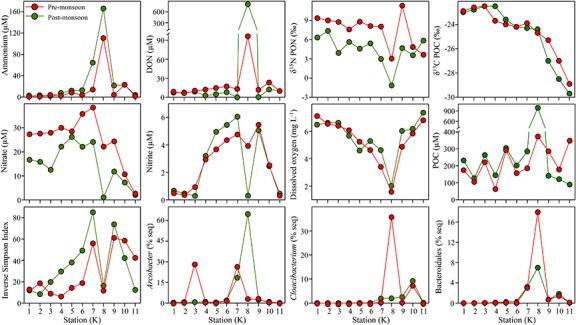
Dynamic of dissolved inorganic and organic nitrogen, particulate organic carbon, stable isotopes in the particulates, dissolved oxygen concentration, diversity, main genera at the sewage and order Bacteroidales. Station K8 was strongly influenced by sewage pollution.

**FIGURE 6:**
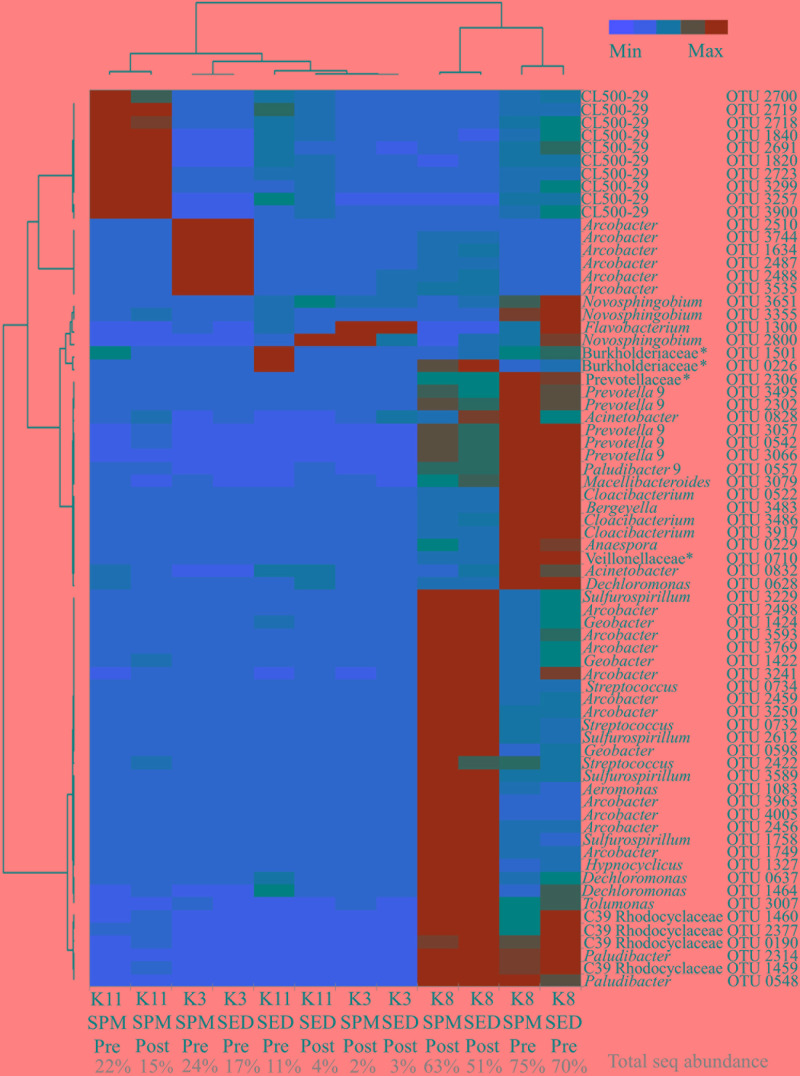
Heatmap of the core OTUs at the sewage (K8) in comparison with K3 and K11. SPM, suspended particulate matter; SED, SPM enriched with sediments; Pre, pre-monsoon; Post, post-monsoon. (*) unclassified.

Depleted values of δ^15^N in PON were evidenced at K8 in both seasons and even negative values during the post-monsoon (Figure 5). The maxima of ammonium, DON and POC, the second lowest concentration of dissolved oxygen, poorer values of the inverse Simpson Index (similar to K1), the lowest concentration of nitrate, and one of the minimum concentrations of nitrite were observed at the sewage during the post-monsoon. The trend in the mentioned parameters was similar during the pre-monsoon. The δ^13^C was not influenced by the sewage and its trend decreases from marine to freshwater stations. The main relations between salinity, temperature, ammonium and POC with other environmental parameters are summarized on Table 2. For example, salinity was positively significantly correlated with δ^13^C and nitrate, ammonium with phosphate and PON, and POC with PON and DOC. It is worth to mention the strong negative correlation between ammonium and δ^15^N. The influence of the sewage pollution was stronger in the water quality parameters during the post-monsoon and coincided with highest relative abundance of *Arcobacter* (Figure 5). *Cloacibacterium* presented the maximum of relative sequence abundance at K8 during the pre-monsoon. The Bacteroidales presented abundance peaks during both seasons. The 70 OTUs of the heatmap (Figure 6) represented more than the 50% of the total sequence abundance at K8. The OTUs found at both seasons were those of the genera: *Paludibacter*, *Dechloromonas*, C39 (Rhodocyclaceae), Prevotellaceae unclassified, *Prevotella* 9, *Macellibacteroides*, *Acinetobacter*, *Tolumonas*, and *Streptococcus*.

## Discussion

### *Vibrio* Dynamic: Culturability and Influence of the Environment

The observed endemic nature of *V. cholerae* populations, predominantly occurring in non-culturable state, is an important aspect of *Vibrio* survival withstanding physicochemical stresses in estuarine water, also reported in previous studies in the Bengal coastline (Alam et al., 2007; Neogi et al., 2012). A non-culturable state is a dormant phase to subsist unfavorable conditions can be easily reverted to an active phase in an appropriate environment or within the host. This study observed that culturable *Vibrio* counts were a good estimator for general *Vibrio* species. Additionally, results of this study showing a lower number of *ctxA*-positive strains after APW enrichment indicated a biasness of the culture method, preferentially inducing the growth of co-existent non-toxigenic populations of *V. cholerae* in coastal waters. Although culturable organisms represent one or two orders of magnitude lower than the total population and that culture methods decrease the number of toxigenic *V. cholerae*, bacterial cultures offer a rough and low-cost estimation of general *Vibrio* with an active metabolism.

The combination of copious amounts of organic matter from sewage and warmer temperatures favored likely the abundance of toxigenic *V. cholerae* during the pre-monsoon. Temperature is one of the most important drivers of the distribution of *Vibrio* and a strong predictor of *Vibrio* outbreaks (Huq et al., 2005; Siboni et al., 2016). Although the impact of sewage on main nutrients (e.g., ammonium and PON) was comparatively higher during the post-monsoon, its effect on *ctxA* abundance was not conspicuous. The detection of *ctxA* gene in estuarine waters, especially nearby the sewage discharge point, highlights the direct association of toxigenic *V. cholerae* with the potential to cause an outbreak. *Vibrio cholerae* has the ability to proliferate rapidly under nutrient pulses and untreated wastewater favors the occurrence of *V. cholerae* in aquatic systems (Gil et al., 2004). Moreover, the expression of pathogenic factors in *V. cholerae* is enhanced by anoxia (Xu et al., 2003) as probably occurred at the station influenced by the sewage outlet.

The ecology of *V. cholerae* strains without the *ctxA* gene is also relevant for human and ecosystem health. These strains may contain other toxins and pathogenic factors such as heat stable enterotoxin, type three-secretion system and cholix toxin (Awasthi et al., 2019). Moreover, they may be linked with the emergence of new pathogenic strains. Outbreaks of diarrhea and skin infections have been reported worldwide caused by *V. cholerae* without the genes to produce cholera toxin (Awasthi et al., 2019; Schwartz et al., 2019). On one hand, sewage pollution signifies toxigenic *V. cholerae* of fecal origin reaching the estuarine environment with the potential to impact on aquatic biota and to survive attached to SPM. On the other hand, this means also that environmental *V. cholerae* strains have the potential to exchange genetic information with toxigenic strains of clinical origin, both metabolically active at elevated temperatures. An environment rich in organic matter favors the horizontal transfer of genes and these settings probably facilitate the evolution of new epidemic serogroups and variant strains of toxigenic *V. cholerae* and *V. mimicus* (Neogi et al., 2019).

The load of organic matter in coastal water can be considered as a key driver behind the seasonal dynamic of non-culturable *Vibrio* across the estuarine and pollution gradient. The relation of non-culturable forms of *Vibrio* with the organic matter was closer during the post-monsoon as indicated by the CCA. *Vibrio* species tend to form biofilms in the SPM and display several enzymes for organic matter remineralization (Neogi et al., 2018; Liang et al., 2019). The particulates offer also protection from predation and unfavorable environmental conditions and sometimes are described as islands sheltering pathogenic organisms in the form of biofilms. The conditions of lower temperatures might have contributed to the non-culturable state of *Vibrio* during the post-monsoon.

A strong effect of salinity and higher temperatures on *Vibrio* OTUs was suggested by the CCA ordination; salinity was also correlated with *Vibrio*-GV, *Vibrio* OTUs and CVCs. *Vibrio vulnificus* and *V. parahaemolyticu*s are generally detected at higher salinities than *V. cholerae* (e.g., Kopprio et al., 2017). Several *Vibrio* species, other than those estimated by the qPCR and CARD-FISH probes, influenced likely the OTU dynamic of this genus. Higher temperatures and salinity may have also a strong effect on *Vibrio* diversity. Most *Vibrio* species need at least some salinity for growth and this environmental factor was described as an important driver of *Vibrio* abundance and distribution (Lara et al., 2009, 2011). Furthermore, the observed abundance peaks of toxigenic *V. cholerae* at stations of intermediate salinities during the pre-monsoon might be related to their better survival potential in this salinity range. Among the halophilic *Vibrio* species, *V. cholerae* has extraordinary potential to tolerate lower salinities in the presence of organic nutrients in aquatic environment (Singleton et al., 1982).

The stations more influenced by the sea were characterized by surface waters with elevated values of δ^13^C, pH and nitrate. An elevated ^13^C signature in the POC is characteristic of the mouth of estuaries (e.g., Thornton and McManus, 1994; Kopprio et al., 2018). The POC enriched in ^13^C at the marine stations may be related to organic matter derived from phytoplankton or microzooplankton. Furthermore, the higher pH at the estuarine mouth may favor also the abundance of bicarbonate ions, which are enriched by about 8 ‰ compared to CO_2_ (Ostrom et al., 1997). Some microalgae such as diatoms and cyanobacteria are able to incorporate bicarbonate ions (Moschen et al., 2009) and may increase the δ^13^C in the POC at the marine stations. A higher pH potentially benefited the abundance of *Vibrio* species, which are generally cultured in alkaline media (e.g., APW). Some *Vibrio* species are able to reduce nitrate using it as an electron acceptor for respiration (Lara et al., 2011), but this process is unlikely under the well-oxygenated waters of the marine stations.

The coastal sediments of the Karnaphuli estuary were detected as important reservoirs for non-culturable *V. cholerae*, *Vibrio* species and organic matter. This is in congruence with previous studies claiming that benthic sediment of coastal zones provides shelter and nutrients for *Vibrio* populations (Vezzulli et al., 2009; Neogi et al., 2018). Coastal storms, particularly cyclones and heavy rains, increase water runoff and the resuspension of sediment together with several microorganisms (e.g., Lara et al., 2009). Usually after cyclones, the rural populations of Bangladesh suffer outbreaks of cholera and we inferred that the cyclone Roanu transported upstream *V. cholerae*, organic nutrients and salt water. There were no differences between the nanoplankton and SPM, most of *Vibrio* were detected as free-living or attached to particles lower than 20 μm. Nanoplankton is the fraction of seston, where the higher abundances of *V. cholerae* are usually observed (Lara et al., 2011; Neogi et al., 2018). However, the role of larger fractions as vector of *V. cholerae* should not be underestimated, *Vibrio* species comprise an important proportion of natural microbiome of zooplankton (Constantin de Magny et al., 2008; Grim et al., 2009).

### Bacterial Community Composition and Potential Roles Across the Estuarine Continuum

The lack of seasonal differences at order, genera or OTU level may be related to the strong estuarine gradient. The Rhodobacterales (class Alphaproteobacteria) and the Oceanospirillales, Vibrionales and Cellvibrionales (Gammaproteobacteria) characterized the marine regions of the Karnaphuli estuary. Certain Alphaproteobacteria and Gammaproteobacteria in general are copiotrophic organisms equipped with a number of tools to degrade polymers and particles (Fuhrman et al., 2015). Large amounts of particulate and dissolved organic matter are characteristic of the watercourses flowing into the Bay of Bengal. Gammaproteobacteria and Alphaproteobacteria dominated the bacterial community composition of the waters of the Bay of Bengal (Ghosh and Bhadury, 2018; Rajpathak et al., 2018; Fernandes et al., 2019; Dhal et al., 2020) and these classes together with Deltaproteobacteria were detected abundantly in sediments of the Sundarbans (Basak et al., 2015). In this study, Deltaproteobacteria were mainly represented by *Geobacter* at K8 with relevance for the arsenic cycle (Gnanaprakasam et al., 2017).

The observed dynamic of Rhodobacterales and Oceanospirillales may reflect that these taxa were early colonizers of the allochthonous organic matter flowing into the Bay of Bengal. The Rhodobacterales are dominant and ubiquitous primary surface colonizer, while the Oceanospirillales are a fast-surface-colonizing group (Dang et al., 2008). According to the last authors, the production of extracellular polymeric substances (EPS) by Rhodobacterales facilitates the settlement of other bacterial communities. *Marivivens* may be responsible of this key role at the Karnaphuli estuary. *Roseobacter* clade CHAB-I-5 lineage may colonize particles derived from freshwater organisms, which suffer a considerable osmotic shock at the mouth of the estuary. Pelagic members of *Roseobacter* have been related to chlorophyll peaks in estuaries (Alonso et al., 2010) and play a major role in processing organic matter derived from phytoplankton (Sarmento and Gasol, 2012; Bakenhus et al., 2017). The POC presented typically an enriched ^13^C signature at the mouth of estuaries and several causes explaining this phenomenon were discussed in the previous section.

*Cyanobium* PCC-6307 was the main cyanobacteria at the freshwater stations. Synechococcales bacteria contribute to more than the 25% of the global photosynthesis (Fuhrman et al., 2015). *Cyanobium* PCC-6307 is characterized by strong bioactive compounds against the growth of other microorganisms (Costa et al., 2015) and may influence the freshwater communities. The Microtrichales, Micrococcales and Frankiales of the phylum Actinobacteria may produce also bioactive compounds and help to remineralize recalcitrant organic matter. Actinobacteria are widely distributed in aquatic and terrestrial systems and play a key role in the production of antibiotics, recycling of nutrients and degradation of complex polymers (Alvarez et al., 2017). The Chthoniobacterales (phylum Verrucomicrobia) and Pirellulales (Planctomycetes) were likely associated with the distribution of *Cyanobium* PCC-6307. According to Bunse and Pinhassi (2017), Verrucomicrobia are related with cyanobacterial blooms due to their diverse enzymes to degrade polysaccharides, while Planctomycetes are scavengers of semilabile dissolved organic matter derived from the bloom.

### Sewage Impact: Bacterial Diversity, Presumptive Functions and Potential Pathogens

*Cloacibacterium* and *Arcobacter* as putative EPS producers, Bacteroidales and depletion of ^15^N were clear indicators of the impact of sewage. The δ^13^C dynamic did not indicate sewage pollution. *Cloacibacterium*, detected abundantly during the pre-monsoon, was isolated originally from municipal wastewater and together with other members of the order Flavobacteriales (*Flavobacterium* and *Bergeyella* detected in this study, phylum Bacteroidetes) are associated with the breakdown of complex organic matter (Bernardet et al., 2002). *Cloacibacterium normanense* may play a key role in phosphate removal (Allen et al., 2006) and produces EPS with flocculant and metal removal properties (Nouha et al., 2016; Wang et al., 2018), processes key for bioremediation or water treatment. Additionally, the production of EPS by *Cloacibacterium* may support its role as early colonizer of anthropogenic organic matter.

*Arcobacter* of the order Campylobacterales (phylum Epsilonbacteraeota) was a marker of sewage pollution and even negative δ^15^N values were detected during the post-monsoon. Similar to *Cloacibacterium*, *Arcobacter* species are known to secrete EPS and to form biofilms (Ross et al., 2001), and these properties give to members of this genus, a central role in processes of bioremediation and colonization of organic matter. Furthermore, several *Arcobacter* species are indicators of high levels of fecal pollution and emerging water-borne pathogens causing gastroenteritis, abortions and bacteremia (Collado et al., 2008; Khan et al., 2017). Certain *Arcobacter* species are able to fix atmospheric nitrogen (Collado et al., 2008) and this process may have been linked with the lower values of δ^15^N at the sewage. However, the fixation of atmospheric nitrogen is very unlikely at the dissolved nitrogen concentrations detected, and ^15^N depletion occurred also during the pre-monsoon. As mentioned in the introduction, there is recent evidence of depleted ^15^N values in POM from heavy-polluted aquatic systems. Under condition of elevated ammonium values and oxygen limitation, there is an accumulation of bacterial biomass with ^15^N-depleted values in POM (Lehmann et al., 2002; Kopprio et al., 2018). Values below −20 ‰ have been reported in ammonia emitted by waste (David Felix et al., 2013). Ammonia from ammonification processes in aqueous solution is transformed to ammonium and its incorporation to bacterial biomass depletes likely the signature of POM.

Nitrate and nitrite may be utilized by some bacteria as electron acceptors under hypoxic conditions at the sewage impacted station. Some bacteria, and particularly the Epsilonbacteraeota (mainly *Arcobacter* and *Sulfurospirillum* in this study), utilize a broad range of electron donors, both organic and inorganic, in absence of molecular oxygen. Low dissolved oxygen concentrations were measured at K8 (∼2 mg L^–1^), anoxic conditions are suspected in the waste-water (previous to be mixed with estuarine waters) or near the sediments at K8. The peak of *Arcobacter* coincided with negligible concentrations of nitrate and nitrite. *Arcobacter* species have been shown to degrade organic compounds with nitrate as electron acceptor (Roalkvam et al., 2015). Furthermore, the families Burkholderiaceae and Rhodocyclaceae (C39 and *Dechloromonas*, order Betaproteobacteriales) were described with denitrifying activity (Cao et al., 2017) and may reduce nitrate and nitrite at K8. The remaining nitrate after this fractionation may be characterized by an elevated ^15^N signature and may have contributed to an increment of δ^15^N of POM at the marine stations.

*Prevotella* 9, Prevotellaceae unclassified, *Macellibacteroides* and *Paludibacter* of the order Bacteroidales (phylum Bacteroidetes) were clear indicators of untreated wastewater and hypoxia during both seasons. Bacteroidales bacteria are used to assess sources of fecal pollution (Cao et al., 2017). For example, *Prevotella*, the main genus of the Bacteroidales in the Karnaphuli estuary, is a fiber fermenter associated with non-industrialized populations whose diet contains more dietary fiber (Chen et al., 2017). Other microbial indicators of wastewater input and hypoxia were *Macellibacteroides, Anaerospora*, Veillonellaceae unclassified, *Streptococcus, Hypnocyclicus* and Synergistaceae unclassified. For instance, *Streptococcus* has been linked to the gut microbiota recovery after *V. cholerae* infection (Hsiao et al., 2014); though the genus comprises important pathogens such as *S. pneumonia* and *S. pyogenes*. Gammaproteobacteria were supported likely by the copious amounts of organic matter at the sewage and intermediate stations and played a key function as early colonizers. Genera of potential pathogens other than *Streptococcus, Arcobacter* and *Vibrio* linked with sewage pollution were *Acinetobacter* and *Aeromonas*.

The proportions of *Vibrio*, *Escherichia-Shigella* and *Enterococcus* in the estuary might have been influenced by the selected primers for 16S rRNA analyses. Despite the higher number of GU and CARD-FISH counts for *Vibrio* at the sewage of the Karnaphuli estuary, the relative sequence abundances of this genus were below the threshold of 1%. A deeper study of *Vibrio* communities requires the use of specific primers like Vib-169F and Vib2-r (e.g., Siboni et al., 2016) but with the consequent loss of information about other organisms. Although culturable coliforms were associated to the sewage, other potential indicators such as *Escherichia-Shigella* and *Enterococcus* were not also parts of the core communities at K8. The V4 primer pair has been described as unsuitable for the detection of enteric pathogens in wastewater (Greay et al., 2019). Overall, the results of bacterial diversity obtained in this study may reflect the real dominance of other communities over *Vibrio*, *Escherichia-Shigella* and *Enterococcus*.

## Conclusion and Outlook

Fecal pollution and warmer temperatures were the deduced drivers of the abundance of toxigenic *V. cholerae*. *Vibrio* outbreaks are expected to increase under climate change scenarios and waste-water management strategies are urgently needed to reduce the risks of *Vibrio* and other potential pathogens linked to sewage. The particulates influenced mostly non-culturable *Vibrio*, while salinity in combination with temperature was mostly related with *Vibrio* diversity. Coastal sediments were an important reservoir for *Vibrio* and organic matter. The protection of wetlands would help to reduce sediment resuspension during coastal storms or cyclones, and contribute with nutrient sequestration.

The island biogeography theory explained the dynamic of some bacterial groups in the Karnaphuli estuary. A few organisms act as early colonizers of allochthonous organic matter (freshwater or anthropogenic) and facilitate the later settlement of other bacteria. The presumptive production of EPS by Rhodobacterales, *Arcobacter* or *Cloacibacterium* may facilitate the mentioned role. The changing conditions of estuaries favor the abundance of early colonizers and the copiotrophic setting of the Karnaphuli estuary promoted the abundance of Gammaproteobacteria. Relevant bacteria for waste water treatment, bioremediation and further biotechnological approaches are already in the estuarine system.

The δ^13^C dynamic was hypothetically driven by planktonic organic matter and pH, while the δ^15^N by microorganisms with a depleted ^15^N signature at the sewage. Nitrate and nitrite were likely used as a substrate for bacterial metabolism, particularly by Epsilonbacteraeota, under the hypoxic conditions of the sewage. Several members of Bacteroidales, as well as culturable coliforms and ammonium, were stronger markers of sewage and hypoxia during both seasons. The use of 16S rRNA diversity allows us only to infer some functions on bacterial communities, further studies from a metagenomic and metatranscriptomic approach will confirm key genes involved on bioremediation and biogeochemical processes and their expression. Moreover, to define a pathogen only with the genus is vague (e.g., *Streptococcus*) and the detection of pathogenic factors is desirable; nevertheless, this study give us an idea about the potential risks.

The coastline of Bangladesh is extremely prone to natural disasters and one of the most vulnerable areas to climate change. Higher temperatures, stronger and more frequent coastal storms or cyclones, salinization of upstream courses, eutrophication-like processes, floods and droughts; in combination with an increasing population will signify considerable challenges for water and food security. Low-cost and adaptive management strategies to sustain a good water quality in the estuary are urgently needed in order to reduce the impact of water-borne bacterial diseases, pollutants and eutrophication. The development of educational programs, strategies to cope natural disasters, sanitation and vaccination campaigns and early warning systems are also crucial to enhance social-ecological resilience.

## Data Availability Statement

16S sequencing data are available at ENA (https://www.ebi.ac.uk/ena/data/view/PRJEB35775).

## Author Contributions

GK, SN, RL, BK, SY, and AG performed the study design. GK, SN, HR, and CA carried out the field, laboratory work and data analyses. The first draft of the manuscript was written by GK and the final version was improved by contributions of all authors.

## Conflict of Interest

The authors declare that the research was conducted in the absence of any commercial or financial relationships that could be construed as a potential conflict of interest.

## Footnotes

1 https://ngs.arb-silva.de/silvangs/

## Supplementary Material

FIGURE S1 Relative sequence abundance of the main orders in the fractions comparing seasons and stations (K).
