# Supplementary figures and images for: Vibrio and Bacterial Communities Across a Pollution Gradient in the Bay of Bengal: Unraveling Their Biogeochemical Drivers
Source: Front Microbiol. 2020 Apr 15;11:594. doi: 10.3389/fmicb.2020.00594 (PMC7174592; doi:10.3389/fmicb.2020.00594)

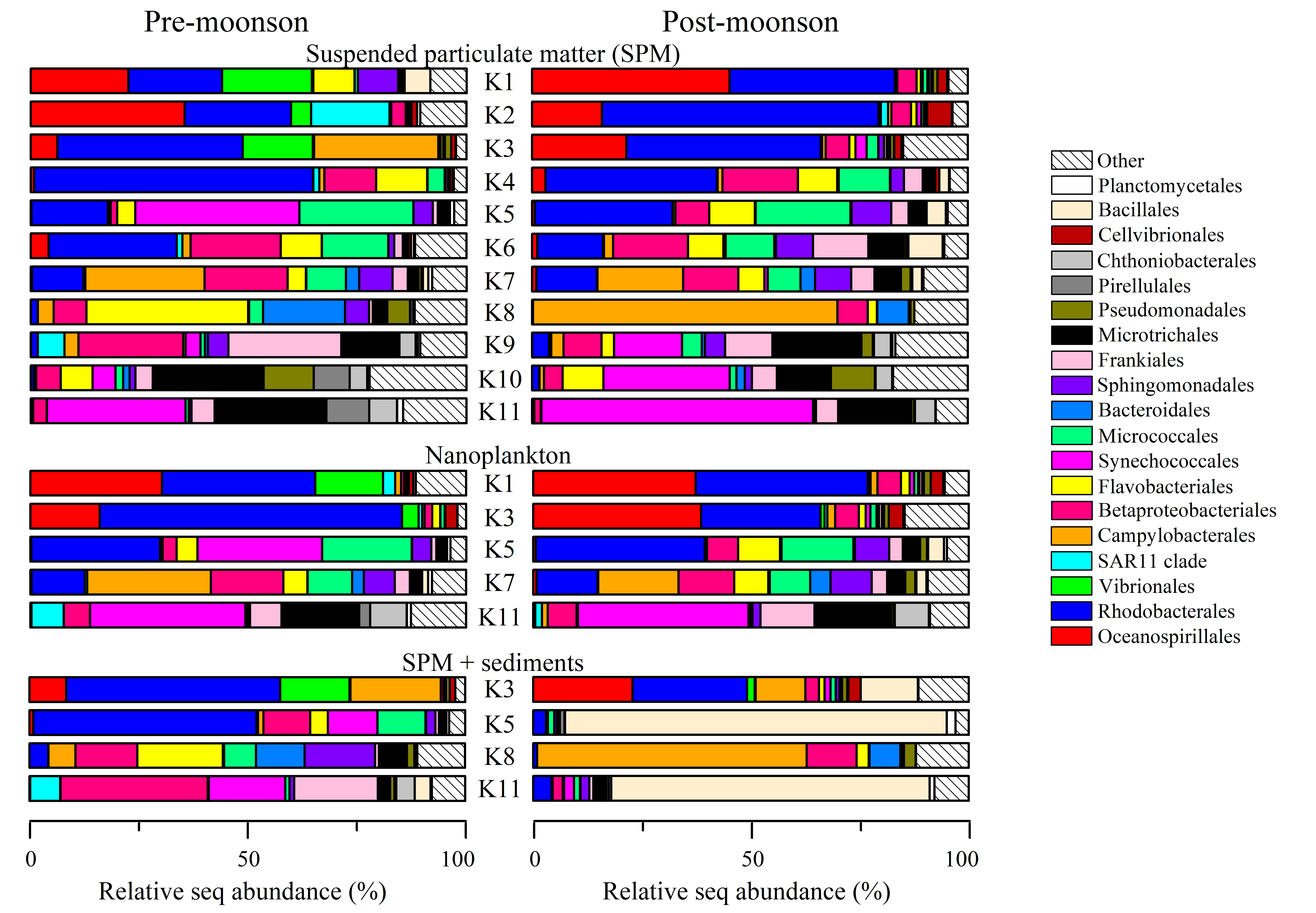

Supplement: FIGURE S1 — Relative sequence abundance of the main orders in the fractions comparing seasons and stations (K). [file Image_1.TIF]
